# *Cdx2* Polymorphism Affects the Activities of Vitamin D Receptor in Human Breast Cancer Cell Lines and Human Breast Carcinomas

**DOI:** 10.1371/journal.pone.0124894

**Published:** 2015-04-07

**Authors:** Claudio Pulito, Irene Terrenato, Anna Di Benedetto, Etleva Korita, Frauke Goeman, Andrea Sacconi, Francesca Biagioni, Giovanni Blandino, Sabrina Strano, Paola Muti, Marcella Mottolese, Elisabetta Falvo

**Affiliations:** 1 Molecular Chemoprevention Unit, Regina Elena National Cancer Institute, Rome, Italy; 2 Department of Epidemiology, Regina Elena National Cancer Institute, Rome, Italy; 3 Department of Pathology, Regina Elena National Cancer Institute, Rome, Italy; 4 Translational Oncogenomics Unit, Regina Elena National Cancer Institute, Rome, Italy; 5 Department of Oncology, Juravinski Cancer Center-McMaster University Hamilton, Hamilton, ON L8V 5C2, Ontario, Canada; 6 Laboratory of Pharmacogenomics (Area Molecular Medicine), Regina Elena National Cancer Institute, Rome, Italy; University of Tennessee, UNITED STATES

## Abstract

Vitamin D plays a role in cancer development and acts through the vitamin D receptor (VDR). It regulates the action of hormone responsive genes and is involved in cell cycle regulation, differentiation and apoptosis. VDR is a critical component of the vitamin D pathway and different common single nucleotide polymorphisms have been identified. *Cdx2* VDR polymorphism can play an important role in breast cancer, modulating the activity of VDR. The objective of this study is to assess the relationship between the *Cdx2* VDR polymorphism and the activities of VDR in human breast cancer cell lines and carcinomas breast patients. *Cdx2* VDR polymorphism and antiproliferative effects of vitamin D treatment were investigated in a panel of estrogen receptor-positive (MCF7 and T-47D) and estrogen receptor-negative (MDA-MB-231, SUM 159PT, SK-BR-3, BT549, MDA-MB-468, HCC1143, BT20 and HCC1954) human breast cancer cell lines. Furthermore, the potential relationship among *Cdx2* VDR polymorphism and a number of biomarkers used in clinical management of breast cancer was assessed in an ad hoc set of breast cancer cases. Vitamin D treatment efficacy was found to be strongly dependent on the *Cdx2* VDR status in ER-negative breast cancer cell lines tested. In our series of breast cancer cases, the results indicated that patients with variant homozygote AA were associated with bio-pathological characteristics typical of more aggressive tumours, such as ER negative, HER2 positive and G3. Our results may suggest a potential effect of *Cdx2* VDR polymorphism on the efficacy of vitamin D treatment in aggressive breast cancer cells (estrogen receptor negative). These results suggest that *Cdx2* polymorphism may be a potential biomarker for vitamin D treatment in breast cancer, independently of the VDR receptor expression.

## Introduction

Vitamin D plays a role in cancer development and acts through the vitamin D receptor (VDR), a nuclear transcriptional factor which belongs to the super family of steroid/thyroid hormone receptors [1–2]. VDR regulates the action of hormone responsive genes and is involved in cell cycle regulation, differentiation and apoptosis [3–4]. Alternative receptors for vitamin D have been recently identified *in vivo*, named ROR ɑ- and ROR γ [5]. This discovery could be helpful in understanding the lack of activity of 20(OH)D_3_ and 20,23(OH)_2_D_3_, typically observed for classical VDR ligands [5].

It has been accepted that *in vivo*, D_3_ metabolism is restricted to its transformation to 25(OH)D_3_ mediated by 25-hydroxylases, subsequent hydroxylation to 1,25(OH)_2_D_3_ by CYP27B1, and degradation mediated by CYP24. However, Slominski et al. [6] recently have provided robust evidences that a novel pathway of D3 metabolism operates *in vivo* that is initiated and regulated by P450scc, modified by CYP27B1, and of which the products and intermediates are biologically active. These products act as partial agonists of the VDR and determinate the translocation of VDR from the cytoplasm to the nucleus with a potency comparable to the 1,25(OH)_2_D_3_ [7].

The active metabolite of vitamin D (1,25(OH)_2_D_3_) plays a key role in maintaining calcium and phosphate homeostasis protecting skeletal integrity, bone mineralization and maintenance of calcium balance. Besides its physiological role, 1,25(OH)_2_D_3_ is a potent inhibitor of breast cancer (BC) cell growth, exerting its anticancer effect through the binding of VDR, which induces the activation of a series of genes involved in cell growth, differentiation and apoptosis [8–9]. Anti-carcinogenic effects of vitamin D in BC may be also mediated via the estrogen pathway by down regulation of the estrogen receptor (ER) [10–11]. It has been hypothesized that a less active VDR, could be associated with either an increased susceptibility to BC risk or a more aggressive disease.

A decrease in VDR protein expression, due to a functional impairment, may be influenced by the polymorphism in the *vdr* gene [12–13]. Over 470 common single nucleotide polymorphisms (SNPs) have been identified in the *vdr* gene and their possible significance in BC has not been fully assessed in epidemiological investigations [14]. These polymorphisms modulate the activity of the *vdr* gene and their frequency differs across multi-ethnic groups. In the Caucasian population, several common allelic variants have been extensively studied in relation to the risk of developing a BC including: i) *Taq1* (located in exon 9 C> T) which leads to a silent codon generation, ii) *Fok1* (located in promoter region 5' of exon 2 C> T), which leads to the synthesis of a longer protein that is less effective as a transcriptional activator of *vdr*; iii) *vdr-5132* (promoter region C> T) which produces a decrease both in the transcriptional activity and in the vitamin D circulating levels; iv) *BMS 1* and *APA* which affect mRNA stability and translational activity of *vdr* gene and v) *Cdx2* (A>G) which significantly alters the transcriptional activity of the *vdr* promoter region [15–18]. Nevertheless, current research results concerning *vdr* gene polymorphisms and BC pathogenesis and progression are still conflicting and the centre of debate [19]. Interestingly, the A allele *Cdx-2* polymorphism, is associated with a significantly higher *vdr* transcriptional activity than the G allele polymorphism. Results from a recent published meta-analysis indicated that individuals who carry variant AA homozygote *Cdx2* had a nearly 16% increased risk of cancer [20]. In the subgroup analysis by ethnicity, results indicated that the association between *Cdx-2* polymorphism and cancer risk is different in Caucasians and African Americans, suggesting genetic diversity among ethnicities [21–23]. Nevertheless, there are limited studies on the relationship between *Cdx2* polymorphism and BC unfavorable biopathological characteristics.

In the present study, we investigated the *Cdx2* polymorphism in the *vdr* gene in a large spectrum of ER-positive (MCF7 and T-47D) and ER-negative (MDA-MB-231, SUM 159PT, SK-BR-3, BT549, MDA-MB-468, HCC1143, BT20 and HCC1954) human breast cancer cell lines. Furthermore, we explored the association between *Cdx2* polymorphism and VDR immunohistochemical expression in an ad hoc retrospective series of 80 human breast carcinomas taking into account breast cancer molecular classification (Luminal A, Luminal B, HER2-Subtype (HS), and Triple Negative) and clinical-pathological parameters routinely detected in breast cancer management.

## Materials and Methods

### Breast cancer cell lines

A collection of ER-positive (MCF7 and T-47D) and ER-negative (MDA-MB-231, SUM 159PT, SK-BR-3, BT549, MDA-MB-468, HCC1143, BT20 and HCC1954), human breast cancer cell lines available at the Regina Elena National Cancer Institute, was analyzed.

Cell lines were cultured as monolayers at 37°C and 5% CO2 in DMEM/F12-GLUTAMAX (Invitrogen-Gibco) supplemented with 10% non-heat inactivated FBS (fetal bovine serum, Invitrogen-Gibco), 5 mg/ml insulin (Sigma-Aldrich) and Hydrocortisone 0.5 μgr/mL (Sigma- Aldrich).

#### Genotyping of Cdx2 polymorphism in breast cancer cell lines

The ER-positive and ER-negative BC cell lines were used to assess the *Cdx2* polymorphism in the vdr gene using pyrosequencing technology as described below. For DNA extraction, cells were lysed in specific buffer (NaCl 300Mm, EDTA 25Mm, Tris 50Mm PH = 8 2% SDS and Proteinase K (IBI Scientific, Peosta, IA) and total DNA was extracted using the kit Phase Lock Gel (PLG-Prime, Gaithersburg, MD) following the manufacturer’s instructions.

#### qRT-PCR analysis

VDR transcript levels were assessed on the same panel of breast cell lines. Total RNA was extracted using the Trizol Reagent (Invitrogen-Gibco, Carlsbad, CA, USA). The first-strand cDNA was synthesized according to the manufacturer’s instructions (M-MLV RT kit, Invitrogen, Carlsbad, CA, USA). Gene expression was measured by real-time PCR using the Fast Sybr-Green assay (Applied Biosystems, Foster City, CA) on a 7900HT instrument (Applied Biosystems, Foster City, CA). Q-PCR primers are listed as follows:

*vdr* F: 5’-GCCCACCATAAGACCTACGA-3’

*vdr* R: 5’-AGATTGGAGAAGCTGGACGA-3’

CYP24A1 F: 5’-GAAAGAATTGTATGCTGCTGTCACA-3

CYP24A1 R: 5’- GGGATTACGGGATAAATTGTAGAGAA-3’

*Beta-actin* F: 5’-GGCATGGGTCAGAAGGATT-3’

*Beta-actin* R: 5’CACACGAGCTCATGTAGAAG-3’

RPL19 F: 5’-CGGAAGGGCAGGCACAT-3’

RPL19 R: 5’-GGCGCAAAATCCTCATTCTC-3’

#### VDR protein detection by Western blot

Total protein extracts were prepared by lysing cells in ice for 30 min in NP40 lysis buffer (50 mM Tris-HCl pH 7.4, 150 mM NaCl, 1% NP-40, 1 mM EGTA, 1 mM EDTA) supplemented with protease and phosphatase inhibitors (5 mM phenylmethylsulfonyl fluoride PMSF, 3 mM NaF, 1 mM DTT, 1 mM NaVO_4_).

All protein extracts were quantified by Bradford assay and equal amounts (30 μg) were loaded onto 8% denaturing SDS polyacrylamide gel electrophoresis (SDS-PAGE), transferred for 2 hours to pure nitrocellulose membrane (Trans-Blot Transfer Medium, Biorad, Hercules, CA). Membranes were blocked in 5% milk-TBS-0.05% Tween 20 for 1 hour and incubated overnight with the indicated primary antibodies. The antibodies antiVDR (C2O) (sc-1008, Santa Cruz Biotechnology, Santa Cruz, CA, USA), anti-p21 waf1/cip1 (#29475, Cell Signalling Technology, Inc. Danvers, MA) and anti-GAPDH (sc-47724, Santa Cruz Biotechnology, Santa Cruz, CA, USA) were diluted in 5% milk-TBS-0.05%. Secondary antibodies were horseradish peroxidase-conjugated (Santa Cruz Biotechnology, Santa Cruz, CA, USA). Signal intensity was quantified using the ECL reagent (Amersham, GE Healthcare, Piscataway, NJ, USA) for the chemo-luminescence detection.

#### Proliferation assay

1,25(OH)_2_D_3_ (Sigma-Aldrich, St Louis, MO, USA) was dissolved according to the manufacturer’s instructions.

To evaluate cell growth, cells were seeded at 20000–50000 cells per 6-well dish and treated or not with different doses of 1,25(OH)_2_D_3_ (25, 50, 100, 200nM). Cells were harvested at the indicated times by trypsin detachment and counted by Z1 Coulter Particle Counter (Beckman Coulter, Fullerton, CA).

#### Colony-forming assay

Cells were seeded at 250–1000 cells per 6-well dish according to their proliferation ability. Formed colonies were stained with crystal violet 10–15 days later and counted. To maintain vitamin D levels constant, the medium was changed every 48 hours in both experiments.

Each experiment was performed in triplicate and repeated at least three times.

#### Wound healing assay

Cells grown to a 85% confluence were seeded in 6-well tissue culture plates and wounded with a sterile 10-μL pipette tip to remove cells. Digital micrographs were taken after scratching and at different times during progression of migration according to characteristics of cells assessed. Each experiment was performed in triplicate and repeated at least three times.

### Patients

To validate the results observed in the breast cancer cell lines, we studied a retrospective series of 80 breast cancer patients collected from the archives of Pathological Department of Regina Elena National Cancer Institute, (Rome, Italy). BC patients included 74 (91%) invasive ductal carcinomas and 6 invasive lobular carcinomas (9%).

Twenty-four BC patients (30%) were T1, 56 (70%) were T2 or T3. Forty-three (54%) BC were graded as well differentiated (G1) or moderately (G2) while 37 (46%) were poorly differentiated (G3) carcinomas. Furthermore, 42 (53%) patients were node positive and 38 (47%) node negative. Estrogen (ER) and Progesterone Receptors (PgR) were positive in 51 (64%) and 34 (42%) BC respectively, and HER2 was positive in 18 (22%) cases.

Tumours were graded according to Bloom and Richardson and staged according to the Unione Internationale Contre le Cancer tumor-node-metastasis system criteria and histologically classified according to the World Health Organization [24], together with *Cdx2* polymorphism and IHC VDR expression, are summarized in Table 1.

**Table 1:** Biopathological characteristics of the 80 Breast Cancer patients.

	N	%
**Number of patients**	80	
**Hystotype**		
Infiltrating Ductal	74	91
Infiltrating Lobular	6	9
**Grading**		
G1+G2	43	54
G3	37	46
**Tumour size**		
T1	24	30
T2+T3	56	70
**Nodal status**		
N0	38	47
N+	42	53
**Estrogen receptor (ER)**		
Positive (≥10%)	51	64
Negative	29	36
**Progesteron receptor (PgR)**		
Positive (≥10%)	34	42
Negative	46	58
**HER2**		
Negative (score 0/1+/2+NA)	62	78
Positive (score 2+A/3+)	18	22
**Ki67**		
Positive (≥15%)	47	59
Negative	33	41
**Molecular Subtypes**		
LA	30	38
LB	17	21
HS	18	22
TN	15	19
**Polymorphism Cdx2**		
GG	42	53
GA	28	35
AA	10	12
**VDR-IRS expression**		
VDR (-)	25	31
VDR (+)	55	69

According to the expression of protein biomarkers, BC were divided into four subtypes: 30 (38%) Luminal A (LA) and 17 (21%) Luminal B (LB), both estrogen (ER) and progesterone (PgR) receptors positive, but characterized by a low and high proliferation index respectively, 18 (22%) HER2 subtype (HS) defined by over expression/amplification of the HER2 gene and lack of hormonal receptor expression and 15 (19%) Triple Negative/basal-like (TN) lacking ER, PgR and HER2 expression. Each patient was informed in advance about the study protocol in both verbally and in writing and provided an informed consent. This study was reviewed and approved by the Ethical Committee of the Regina Elena National Cancer Institute (Prot. CE/494/12).

#### Immunohistochemistry

In the 80 cases included in the study, the ER and PgR status as well as HER2 and Ki-67 were assessed by immunohistochemistry (IHC) on formalin-fixed paraffin-embedded tissues (FFPE) by using the monoclonal antibodies (MoAbs) 6F11, 1A6 (Menarini, Florence, Italy), the polyclonal antibody A0485 (Dako, Milan, Italy) and the MoAb MIB-1 (Dako), respectively. VDR expression was analyzed using the MoAb 2F4 (Abd Serotec, Space, Milan, Italy). Two micron-thick sections were stained with a streptavidin-enhanced immunoperoxidase technique (Supersensitive Multilink, Menarini) in an automated autostainer (Bond Max, Menarini) using a pH 6 citrate buffer antigen retrieval protocol for all the antibodies used throughout the study.

#### Scoring criteria

HER2 was scored as 0 and 1+ as negative, 2+ equivocal (to be confirmed by ISH test), and 3+ positive on the basis of Wolff et al. [25].

ER and PgR were considered positive when ≥10% and Ki-67 when ≥15% of the neoplastic cells showed distinct nuclear immunoreactivity.

VDR score was assigned according to Remmele and Stegner [26]. The assessment of the degree of staining and distribution patterns of specific immunohistochemical staining were evaluated using a semi-quantitative assay. The IRS was calculated by multiplication of the staining intensity. The percentage of cells with positive staining was scored as follows: 0 = no, 1 = weak, 2 = moderate, and 3 = strong staining; the percentage of positively stained cells was scored as follows: 0 = no staining, 1 = <10% of cells, 2 = 11% to 50% of cells, 3 = 51% to 80% of cells, and 4 = >81% of cells stained. The total score per sample therefore ranged from 0 to 12; for purposes of analyses we categorized the scores as follow: 0 to 4 indicates a negative staining while 6 to 12 indicates positive staining. Evaluation of the IHC results, blinded to all patient data, was performed independently by two investigators (MM, ADB).

#### Genotyping of Cdx2 polymorphism in human breast carcinomas

In the 80 cases included in the study, genomic DNA from breast tumour frozen tissue (50–100 mg) was extracted using Trizol (Invitrogen, Carlsbad, CA) following manufacturer’s instructions.

The concentration and purity of total DNA were assessed using a Nanodrop 1000 spectrophotometer (Nanodrop Technologies, Wilmington, DE, USA). The concentration and purity of total DNA were assessed using a Nanodrop 1000 spectrophotometer (Nanodrop Technologies, Wilmington, DE, USA).

The polymorphism of the gene VDR receptor *Cdx2* (rs 11568820) was evaluated.

PCR reaction for this polymorphic gene was performed as Real Time PCR using Rotorgene Instrument (Corbett Life Science, Concorde, NSW, Australia) following PCR (Polymerase Chain Reaction) conditions provided by the manufacturers. The ad-hoc primers for *Cdx2*, were designed as described below:

Primer F: 5’-ATGGGCTGTGAAATAAATTTGGT-3’

Primer R biot: 5’-CTTCCCAGGACAGTATTTTTCAA-3’

PCR reactions was carried out in a volume of 50 μl containing: 10 mM Takara deoxynucleotide triphosphate (dNTP) mixture, 20 pmol primers, approximately 30–40 ng of DNA template, Takara 5XR-PCR Buffer (Mg^2+^free), Takara 50mM Mg^2+^, EvaGreen Dye 20X and Takara Ex TaqR-PCR Custom (5U/ul). Reaction condition was as follows: initial denaturation at 95°C for 3 min, then 35-cycles of denaturation at 95°C for 30 sec; annealing at 62°C for 30 sec; elongation at 72°C for 30 sec and final extension at 60°C for 5 min, then 5-cycles of green channel signal acquisition at 60°C for 30 sec. PCR products were evaluated on 2,5% agarose gel (Bio-Rad, Milano, Italy), stained with ethidium bromide (Sigma-Aldrich, St Louis, MO, USA). In addition, one control for each genotype was generated.

The target sequence containing the polymorphic site was amplified using standard PCR conditions Specific sequence primer was used for Cdx2 as described below:

Primer S: 5’-ATTCCTGAGTAAACTAGGTC-3’

The polymorphic gene was analyzed using Pyrosequencing technologies (instrument PyroMark MD-Biotage, Uppsala, Sweden) according to a previously published method [27].

#### Statistical analysis

Descriptive statistics were used to summarize pertinent study information. Variables were reported as frequencies and percentage values. The Chi-Square test and Fisher exact text, when appropriate, were used to assess the relationship between *Cdx2* and all the bio-pathological parameters (ER, PgR, HER2, Ki-67, p53 level, tumour size, grading, nodal status, histotype). We also investigated the relationship between *Cdx2* and VDR expression. Multiple correspondence analysis (MCA), a descriptive/exploratory technique designed to analyze simple two-way and multi-way tables, was used to identify prognostic biological profiles. The results provide information that is similar in nature to that produced by factor analysis techniques and make it possible to explore the structure of categorical variables included in the table. The most common kind of this type is the two-way frequency cross-tabulation table [28]. This representation aims to visualize the similarities and/or differences of profiles, simultaneously identifying those dimensions that contain the majority of the data variability. The positions of the points in the MCA graph are informative. Categories plotted close to each other are statistically related and are similar with regard to the pattern of relative frequencies. *Cdx2*, ER, PgR, HER2, Ki67, tumour size (T), lymph node status (N), and histological grade [G]) were the variables of major interest for the purpose of our study. These factors were introduced in each analysis as active variables.

P-values < 0.05 denotes statistically significant associations. Statistical analyses were carried out using SPSS software (SPSS version 21.0, SPSS Inc., Chicago, Illinois, USA).

## Results

### Analysis of *Cdx2* polymorphism in human breast cancer cell lines

We investigated the *Cdx2* status in 2 ER-positive (MCF7 and T-47D) and in 8 ER-negative (MDA-MB-231, SUM 159PT, SK-BR-3, BT549, MDA-MB-468, HCC1143, BT20 and HCC1954) human breast cancer cell lines by pyrosequencing analysis. The *Cdx2* genotyping was differently distributed among the cell lines tested: AG for MDA-MB-231, SUM 159PT; GG for HCC1143, BT20 and HCC1954 and AA for SK-BR-3, BT549 and MDA-MB-468 and AG for MCF7 and T-47D (Table 2).

**Table 2:** *Cdx2* status in human breast cancer cell lines by ER status.

Breast Cancer cell lines	Cdx2 Polymorphism		
	AG	GG	AA
**ER (+)** ^**a**^			
MCF7	•		
T47D	•		
**ER(-)** ^**a**^			
MDA-MB-231	•		
SUM 159PT	•		
SK-BR-3			•
BT549			•
MDA-MB-468			•
HCC1143		•	
BT20		•	
HCC1954		•	

^a^Estrogen receptor.

### Analysis of *vdr* and CYP24A1 mRNA transcript and VDR protein basal levels in breast cancer cell lines

**Fig 1:**
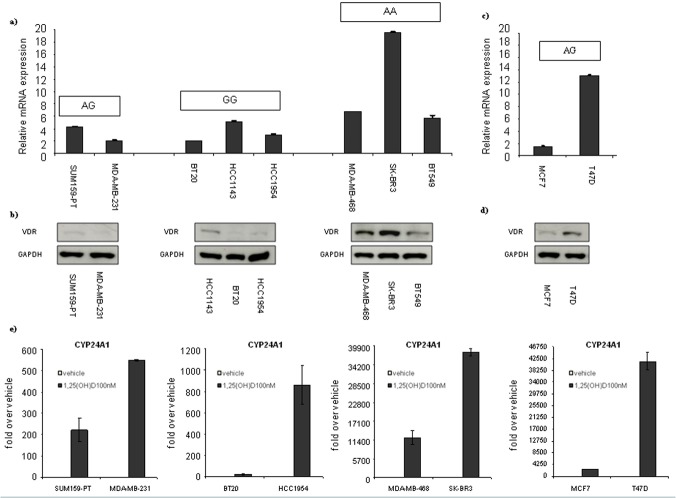
VDR transcript and protein basal levels. (a, c) Q-PCR for the expression of VDR gene from a panel of breast cancer cell lines. (b, d) Representative western blot analysis of whole cell lysates obtained from breast cancer cell lines stained with the indicated antibodies. GAPDH staining was used as a loading control. Cells were separated based on their *Cdx2* status. (e) Q-PCR for the expression of CYP24A1 gene from eight representative breast cancer cell lines treated or not with 100 nM of 1,25(OH)2D_3_ for 24 hours.

We evaluated the relative *vdr* mRNA and VDR protein expression in the 10 BC cell lines previously genotyped for *Cdx2* polymorphism (Fig 1). ER(-) BC cell lines characterized by the genotype AA (SK-BR-3, BT549, MDA-MB-468) have a higher level of both *vdr* transcript and VDR protein than BC cell lines presenting an AG or GG genotype (Fig 1, panels a and b). In the 2 ER(+) BC cell lines both displaying a AG genotype, the T47D showed a higher mRNA transcript and protein level than MCF7 cell line (Fig 1, panels c and d). Indeed, we evaluated the VDR activity after 1α,25(OH)2D_3_ treatment in eight representative breast cancer cell lines. For this reason we measured the mRNA levels of a well known VDR direct target, CYP24A1 [7,29]. This target is implicated in the 1α,25(OH)_2_D_3_ degradation and it was found to negatively correlate with a good prognosis in several types of cancer. Recently, CYP24A1 was found to be an interesting marker of melanocytic nevi formation and melanomagenesis [30].

CYP24A1 mRNA levels results upregulated in all the cell lines treated for 24 hrs with 1α,25(OH)2D_3_ compared to the untreated ones. mRNA folds induction resulted higher in ER(+) BC cell lines (MCF-7, T47D) and ER(-) BC cell lines characterized by the genotype AA (SK-BR-3, MDA-MB-468) BC cell lines compared to the other ER(-) BC cell lines tested (Fig 1, panel e).

### Vitamin D anticancer effect

In order to evaluate whether the vitamin D treatment correlates with the *Cdx2 s*tatus and presence/absence of ER, we selected 6 representative ER(–) breast cancer cell lines characterized by different polymorphisms: AA (SK-BR3 and MDA-MB-468), AG (MDA-MB-231 and SUM159PT), GG (BT20 and HCC1954) and two ER(+) breast cancer cell lines with AG status of *Cdx2* (MCF7 and T47-D).

**Fig 2:**
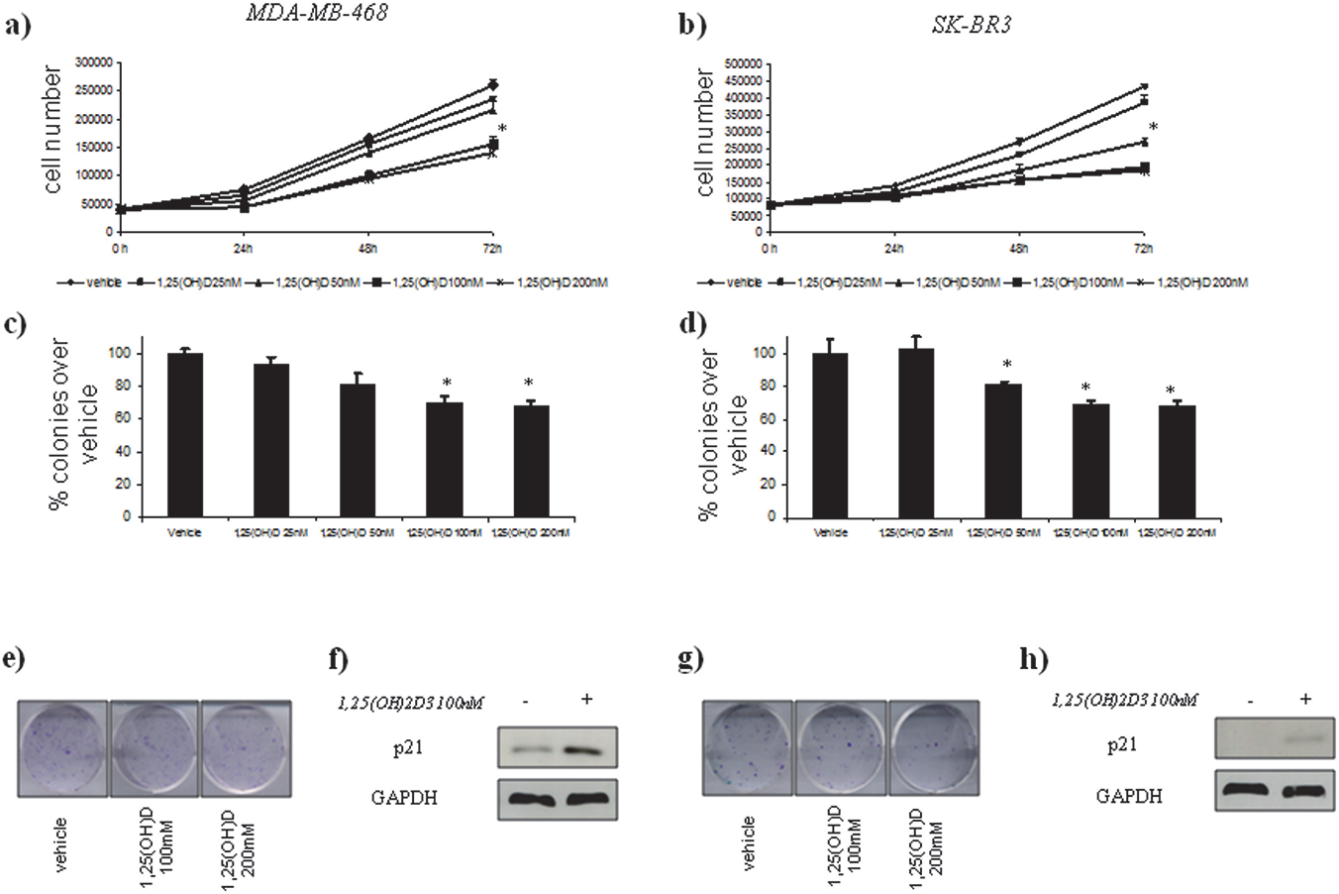
Vitamin D anticancer effects in vitro on ER(–) breast cell lines with AA *Cdx2* status. Time-dependent growth curves of MDA-MB-468 (a) and SK-BR3 (b) cells in the absence or in the presence of different doses of 1,25(OH)2D_3_ (25–200 nM). Histograms (c and d) showing average colony percentage over vehicle from duplicate experiments at 10–21 days from seeding. Bars indicate the average of three independent experiments. * statistics: p < 0.05. Representative micrographs of colonies formed by MDA-MB-468 (e) and SK-BR3 (g) cells treated with 1,25(OH)_2_D_3_ as indicated. Representative western blot analysis of whole cell lysates obtained from MDA-MB-468 (e) and SK-BR3 (f) cells treated as suggested and stained with the indicated antibodies. GAPDH staining was used as a loading control.

We observed an inhibition of the cell growth and colony forming ability of the treated SK-BR3 and MDA-MB-468 cells. (Fig 2, panels a, b, c, d). To establish whether 1,25(OH)_2_D_3_ triggers the activation of tumour-suppressor pathways, we performed a western blot analysis of p21 protein levels, a major transcription target of activated p53, after vitamin D treatment. We found an increased in p21 protein levels after vitamin D treatment in SK-BR3 and MDA-MB-468 cell lines (Fig 2, panels f and h).

**Fig 3:**
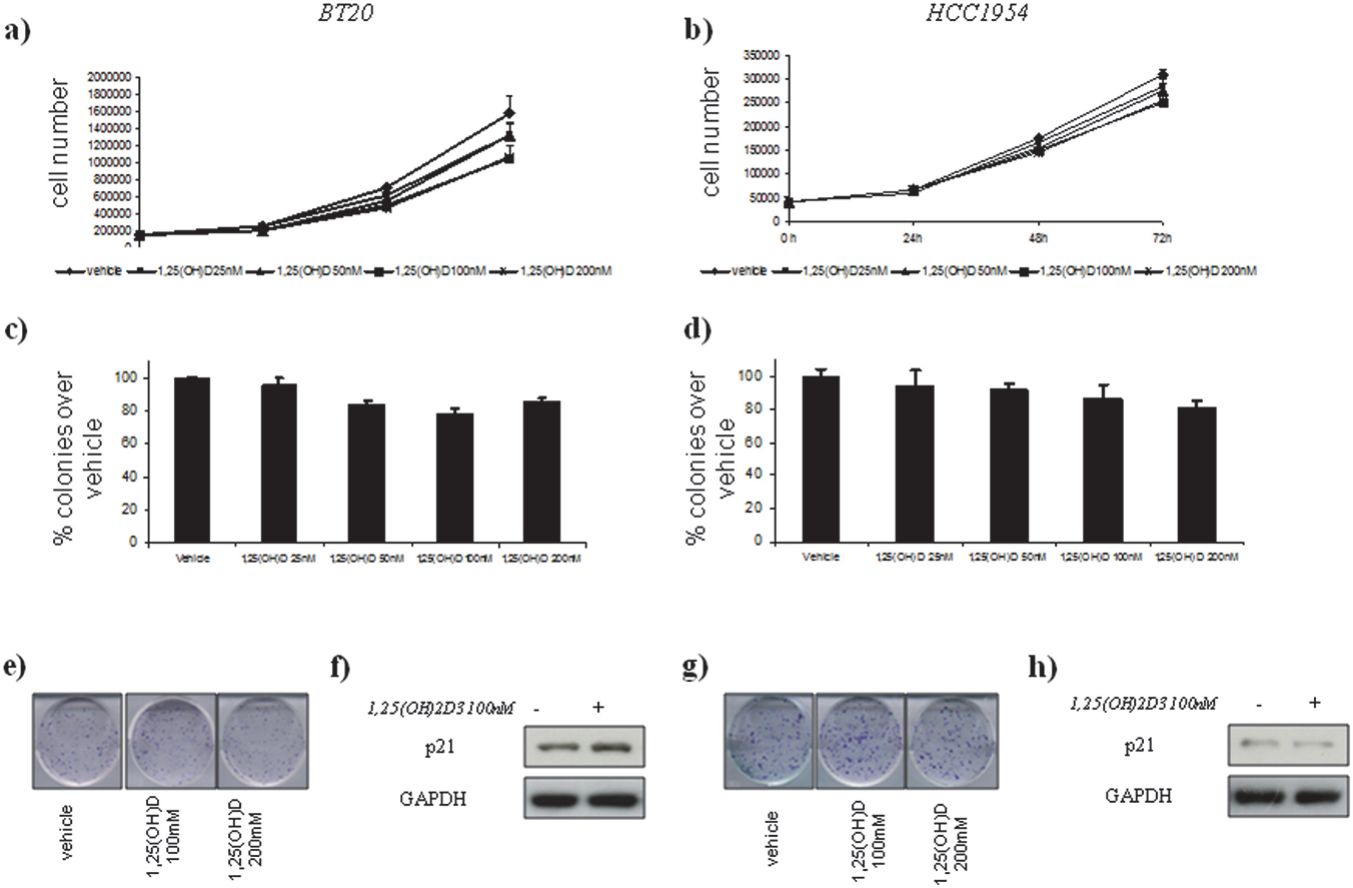
Vitamin D anticancer effects in vitro on ER(–) breast cell lines with GG *Cdx2* status. Time-dependent growth curves of BT20 (a) and HCC1954 (b) cells in the absence or in the presence of different doses of 1,25(OH)2D_3_ (25–200 nM). Histograms (c and d) showing average colony percentage over vehicle from duplicate experiments at 10–21 days from seeding. Bars indicate the average of three independent experiments. Representative micrographs of colonies formed by BT-20 (e) and HCC1954 (g) cells treated with 1,25(OH)_2_D_3_ as indicated. Representative western blot analysis of whole cell lysates obtained from BT20 (e) and HCC1954 (f) cells treated as suggested and stained with the indicated antibodies. GAPDH staining was used as a loading control.

**Fig 4:**
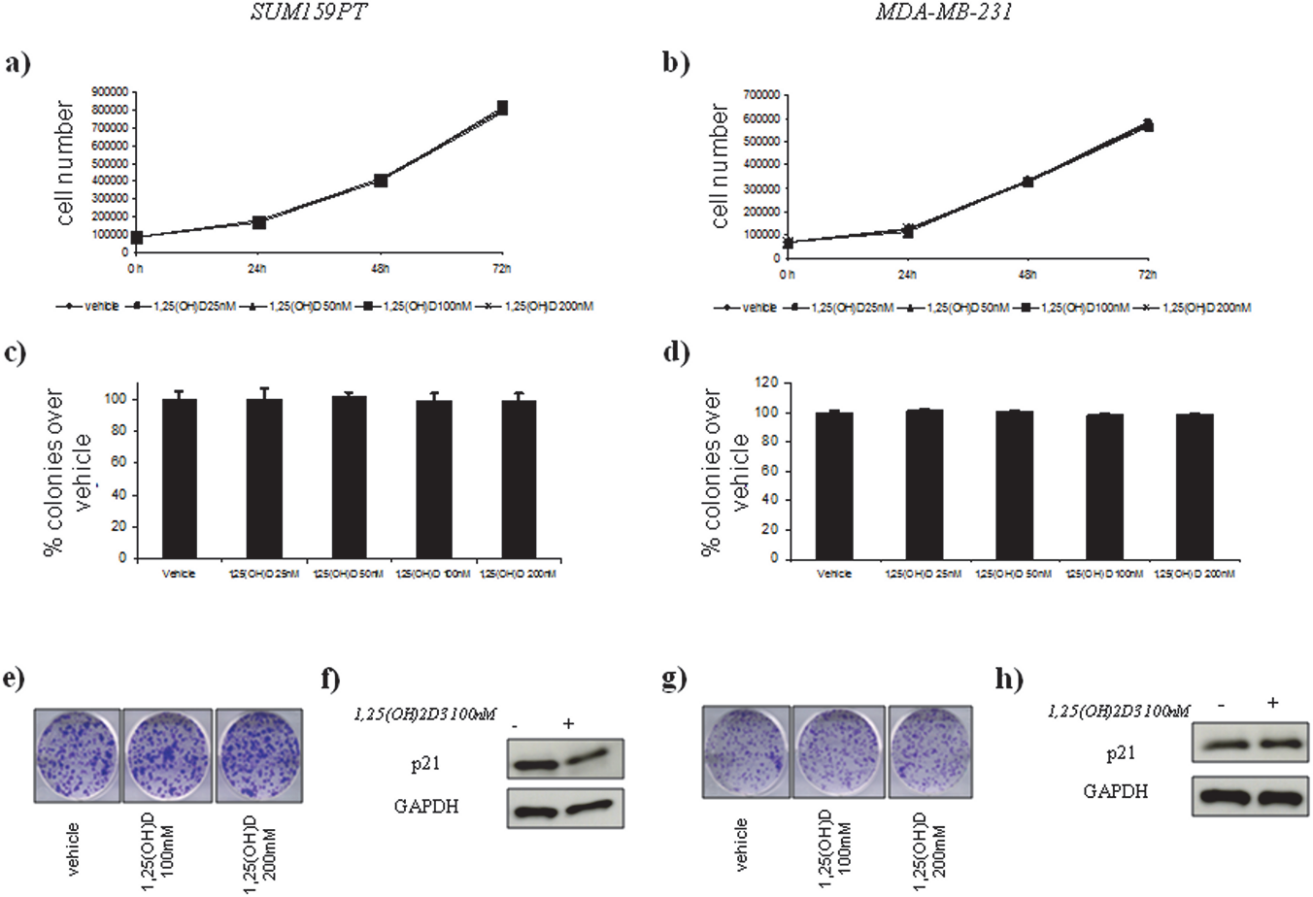
Vitamin D anticancer effects in vitro on ER(–) breast cell lines with AG *Cdx2* status. Time-dependent growth curves of SUM159PT (a) and MDA-MB-231 (b) cells in the absence or in the presence of different doses of 1,25(OH)2D_3_ (25–200 nM). Histograms (c and d) showing average colony percentage over vehicle from duplicate experiments at 10–21 days from seeding. Bars indicate the average of three independent experiments. Representative micrographs of colonies formed by BT-20 (e) and HCC1954 (g) cells treated with 1,25(OH)_2_D_3_ as indicated. Representative western blot analysis of whole cell lysates obtained from SUM159PT (e) and MDA-MB-231 (f) cells treated as suggested and stained with the indicated antibodies. GAPDH staining was used as a loading control.

Instead, vitamin D did not affect cell growth and colony forming ability of the treated BT20 and HCC1954 treated cells (Fig 3, panels a,b,c,d). and SUM159PT and MDA-MB-231 (Fig 4, panels a,b,c,d).

**Fig 5:**
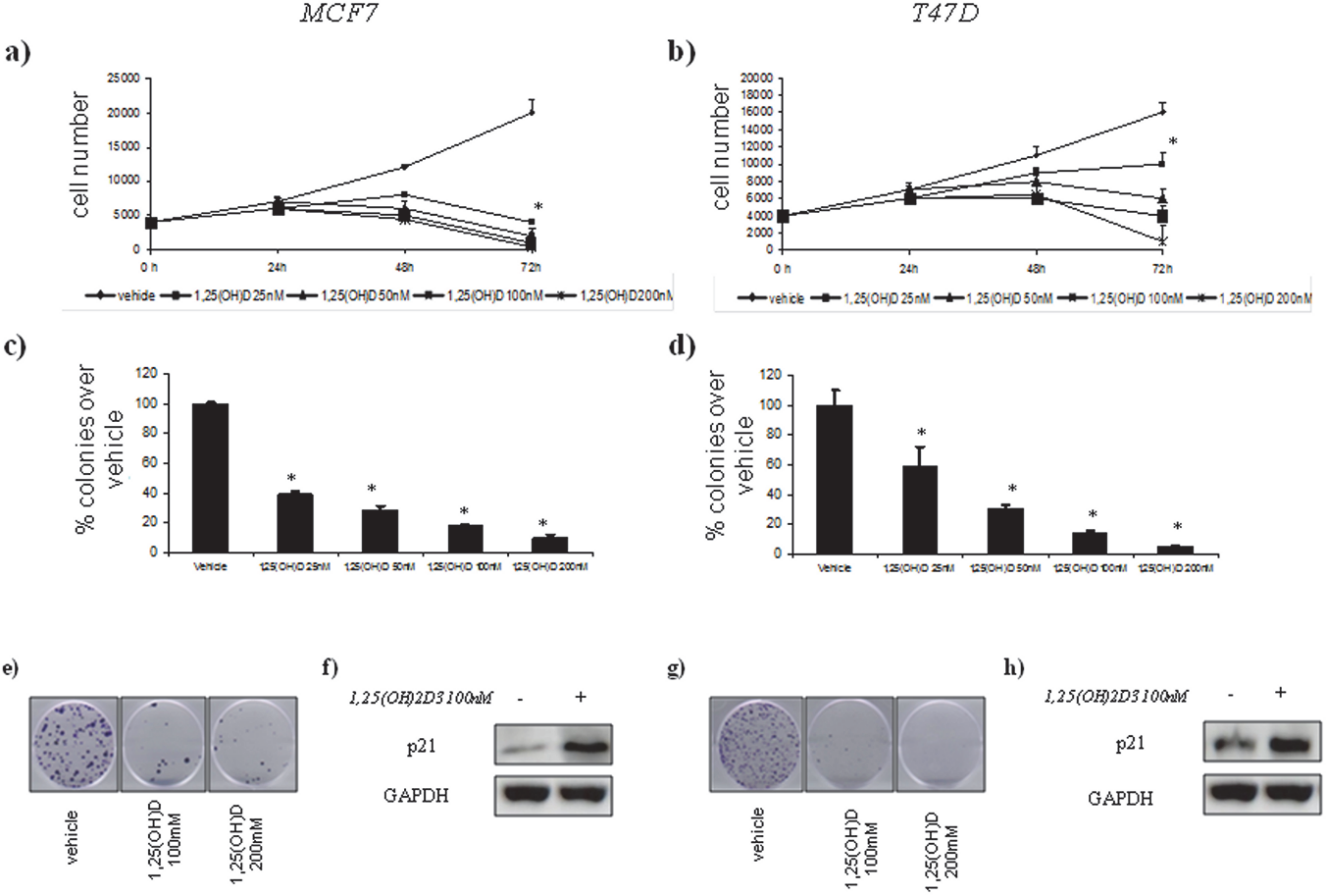
Vitamin D anticancer effects in vitro on ER(+) breast cell lines with different *Cdx2* status. Time-dependent growth curves of MCF7 (a) and T47D (b) cells in the absence or in the presence of different doses of 1,25(OH)2D_3_ (25–200 nM). Histograms (c and d) showing average colony percentage over vehicle from duplicate experiments at 10–21 days from seeding. Bars indicate the average of three independent experiments. * statistics: p < 0.05. Representative micrographs of colonies formed by BT-20 (e) and HCC1954 (g) cells treated with 1,25(OH)_2_D_3_ as indicated. Representative western blot analysis of whole cell lysates obtained from MCF7 (e) and T47D (f) cells treated as suggested and stained with the indicated antibodies. GAPDH staining was used as a loading control.

Moreover, 1,25(OH)_2_D_3_ treatment affected cell growth and colony formation of the ER(+) breast cancer cell lines (MCF7 and T47-D) (Fig 5, panels a, b, c, d). We found an increased in p21 protein levels after vitamin D treatment in MCF7 and T47D cell lines (Fig 5, panels f and h).

**Fig 6:**
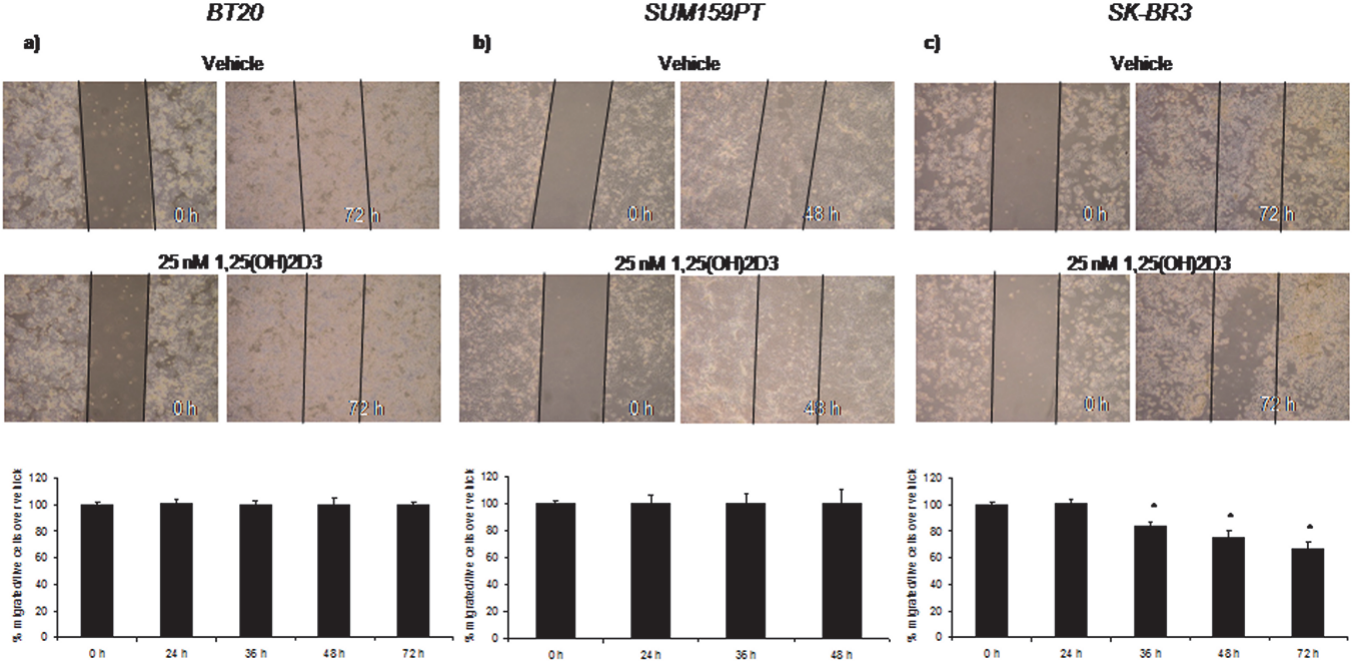
Vitamin D wound healing assay. Upper part: representative micrographs of wound healing closure assays from BT20 (a), SUM159PT (b) and SK-BR3 (c) cells, treated as indicated for 48–72 hrs. Lower part: histogram showing the healing closure efficiency of the cells treated with vehicle or 25 nM 1,25(OH)2D_3_ at the indicated times. Bars indicate the average of three independent experiments. * p-value < 0.05.

Based on these observations, we hypothesized that vitamin D might differently affect the migratory capability of ER(–) breast cancer lines according to *Cdx2 status*. Wound-healing assay vitamin D treatment revealed a time-dependent effect of vitamin D on the migration into the wound of SK-BR3 (AA) cells at low dose (25 nM) (Fig 6, panel c, top and down), while no changes were observed in BT20 (GG) and SUM159PT (AG) cells (Fig 6, panels a and b, top and down).

### Relationship between *Cdx2* polymorphism and biopathological parameters

**Fig 7:**
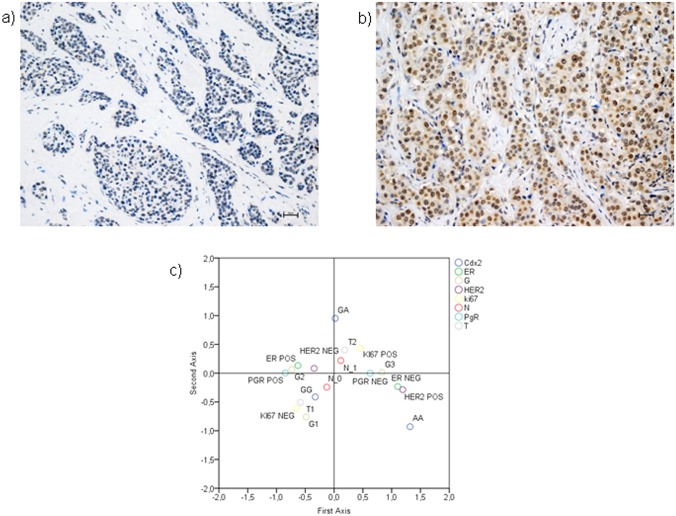
Immunohistochemical analyses. Representative immunohistochemical figures of ductal infiltrating breast cancer with low VDR expression (score 3) (a) and with high VDR expression (score 8) (b). Scale bar 30 μ. (c) The MCA graph demonstrated that phenotype AA is located in the quadrant containing the most aggressive parameters (T3 tumours, HER2 positive, ER negative and G3 in contrast to phenotype GG which is associated to more favorable bio-pathological parameters [T1 tumours, N0, G1, ki67 (-)].

As shown in Table 2, the GG, GA and AA phenotypes are present in 42 (53%), 28 (35%) and 10 (12%) tumours, respectively. The observed allele frequencies of the gene polymorphisms were comparable to those reported for European populations in the dbSNP database. (https://www.ncbi.nlm.nih.gov/SNP/). VDR was positive, with a variable degree of intensity (see Immunohistochemistry paragraph), in 55 (69%) cases. Of these, 17 (31%) were moderately positive (score 6–8) and 38 (69%) were highly positive (score 9–12) [31]. VDR displays a prevalently nuclear positivity, as reported in Fig 7A. As shown in Fig 7B, the complex interrelationship among the bio-pathological variables considered in our study can be better evaluated by using the MCA. This analysis permits to graphically explore the associations among pathological (T, G, N), and biological variables (ER, PgR, HER2, Ki-67) visualizing their link with the *Cdx2* polymorphism. The graphic shows that the AA genotype is associated with ER negative, HER2 positive, G3 and T3 BC (upper left quadrant) while the GG genotype is associated to a less aggressive BC profile (G1, T1, low Ki-67, HER2-, PgR+, N0) (upper right quadrant).

### Relationship between *Cdx2* polymorphism and VDR expression

Table 3 summarizes the relationship between *Cdx2* polymorphism and BC molecular subtypes. Patients with the AA genotype are more likely to be a HS or TN (50% and 40%, respectively), while patients with GA and GG genotypes are more likely to be a LA or LB (40 and 43%, respectively) resulting in these differences being significant (p = 0.05).

**Table 3:** Association between polymorphism Cdx2 and Molecular subtypes.

Cdx2	LA	LB	HS	TN	TOTAL
AA	1 (10%)	0 (0)	5 (50%)	4 (40%)	10 (100%)
GA	11(40%)	6(21%)	5 (18%)	6 (21%)	28 (100%)
GG	18 (43%)	11 (26%)	8 (19%)	5 (12%)	42 (100%)
Total	30 (100%)	17 (100%)	18 (100%)	15 (100%)	80 (100%)

P-value (Chi-Square test) = 0.05.

In particular, when we analyzed the distribution of *Cdx2* according to ER status (Table 4), we observed that 9 BC out of 10 (90%) with genotype AA were ER negative. ER positive BC more frequently presented a GA (64%) or GG (76%) genotype (p<0.0001).

**Table 4:** Association between polymorphism Cdx2 and ER status.

Cdx2	ER(-)	ER(+)	TOTAL
AA	9 (90%)	1 (10%)	10 (100%)
GA	10 (36%)	18 (64%)	28 (100%)
GG	10 (24%)	32 (76%)	42 (100%)
Total	29 (36%)	51 (64%)	80 (100%)

P-value (Chi-Square test) < 0.0001.

Conversely, VDR expression is equally distributed across the 4 different molecular subtypes (p = 0.752, data not shown) When we explored the relationship between *Cdx2* polymorphism and VDR expression, we observed that the AA is low levels of VDR in the majority of cases (80%) whereas the GA and GG genotypes are more likely to express VDR (75% and 76%, respectively, p = 0.002).

**Fig 8:**
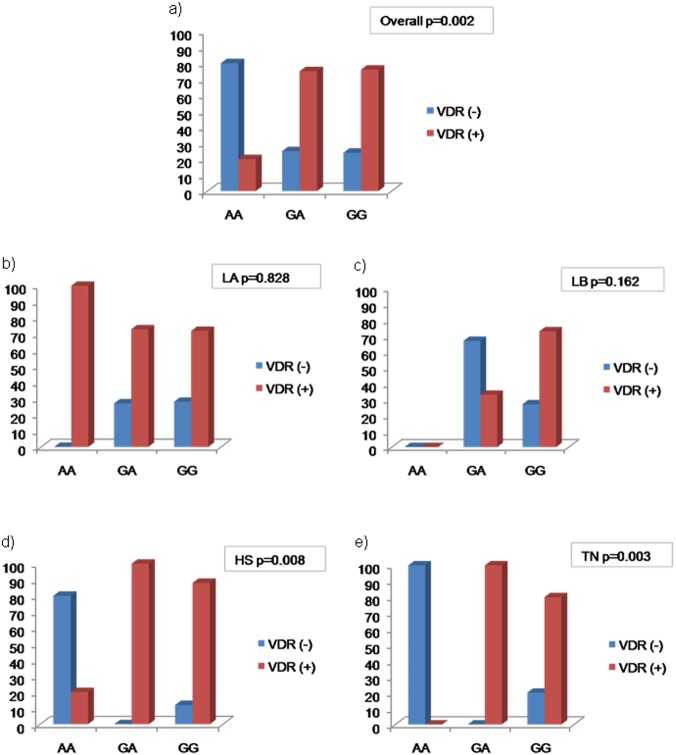
Relationship between polymorphism *Cdx2* and VDR expression in 80 BC cases and according to molecular subtype. VDR is differently expressed in *Cdx2* phenotype (a). There is no difference in VDR expression according to *Cdx2* phenotype in LA (b) and LB (c). In contrast, in HS (d) and in TN (e) BC, VDR is more likely to be expressed in GA and GG phenotypes.

When we stratified our BC series by molecular subtypes, the association between *Cdx2* polymorphism and VDR expression is maintained only in HS and TN (p = 0.008 and p = 0.003, respectively), but not in LA and LB (p>0.05) tumours characterized by ER positivity highlighting the influence of the receptor in this relationship (Fig 8).

## Discussion

Epidemiological observations strongly support the concept of the vitamin D system contributing to the pathogenesis and prognosis of different human cancers, including breast cancer [32–34]. Breast cancer is generally characterized by estrogen-dependent growth, and the proliferative actions of estrogen are mediated via ER. Several studies have established that 1,25(OH)_2_D_3_ modulates cell cycle progression, differentiation, invasion and apoptosis of breast cancer cells. In particular, this evidence has shown that vitamin D suppresses cell proliferation of ER(+) breast cancer cell lines more effectively compared to ER(–) cells [11,35–37]. Moreover, vitamin D analogs do not provide any significant cell inhibitory activity in MDA-MB-231 cells, whereas these vitamin D analogs are antiproliferative in ER (+) cells [10,38].

To our knowledge, it is the first time a study has investigated the correlation between the effect of vitamin D on expression and functional capabilities of VDR focusing on *Cdx2* genotype in a large series of ER-positive and ER-negative human breast cancer cell lines.

The analysis of *Cdx2* polymorphism across our panel of breast cancer cell lines evidenced the presence of different *Cdx2* genotypes: AG in MDA-MB-231, SUM 159PT, MCF7 and T-47D, GG in HCC1143, BT20 and HCC1954 and AA in SK-BR-3, BT549 MDA-MB-468. Interestingly, the ER+ cell lines, MCF7 and T-47D, both presented an AG genotype.

We did not observe any correlations between *Cdx2* status, VDR mRNA and protein levels in the 2 ER(+) cell lines, whereas a correlation between VDR mRNA, protein levels and *Cdx2* genotypes was found in the ER(–) cell lines. In agreement with other reported papers [10,37–38], SKBR3, MDA-MB-468 and BT549, characterized by AA *Cdx2* status, showed a higher level of VDR in comparison to the other 5 ER(–) breast cancer cell lines. In particular, the 2 ER(-) cell lines MDA-MB-231 and SUM 159PT cells showed very low levels of VDR. Moreover, we tested the VDR activity in eight of the ten BC cell lines described before. In particular, we measured the mRNA levels of a direct target of VDR, CYP24A1, after 24 hrs of treatment with 100mM of 1,25(OH)_2_D_3_. All the cell lines showed an upregulation of CYP24A1 mRNA levels, anyway, in MCF-7and T47D, ER (+) cell lines, and in SK-BR3 and MDA-MB-468, ER (-) cell lines with AA *Cdx2* status, we found a strong upregulation of this target compared to the other cell lines. We also demonstrated that the 1,25(OH)_2_D_3_ is effective on ER(+) cells, MCF7 and T47D, in agreement with data previously reported [38]. Additionally, we have found that vitamin D impinges on viability and colony forming ability of SK-BR3 and MDA-MB-468 cells, which are characterized by an AA *Cdx2* status, while no effects have been observed in other ER(–) breast cancer cell lines with AG and GG *Cdx2* status. As well, our data suggested that the efficacy of vitamin D treatment is strongly dependent on the *Cdx2* and VDR status. These data confirmed those reported by Elstner et al. [37], which demonstrated that the ER(-) SK-BR3 cell line is more sensitive to the treatment with analogous of vitamin D than MDA-MB-231 and BT20 cells. Wound healing inhibition assays revealed, also, an effect of vitamin D on the migration of SK-BR3 cells, while it did not affect the migration ability of SUM159PT and BT20 cell lines.

Furthermore, according to Flanagan et al., we found that SUM159PT (AG *Cdx2* status) cell line is characterized by low VDR basal expression and 1,25(OH)2D_3_ treatment did not affect cell proliferation after 72 hrs of treatment [39].

Our data suggest that this genetic variant of VDR receptor (*Cdx2*) may be related to ER-negative breast cancer. Indeed, we found a new association between the *Cdx2* polymorphism and response to 1,25(OH)_2_D_3_ treatment. In particular, ER(-) breast cancer cells with *Cdx2* genotype AA are more responsible to vitamin D compared to cells with genotype AG/GG.

In conclusion, our data on cultured cells suggest that VDR *Cdx2* status could be a marker for the use of vitamin D in the treatment on ER(–) breast cancer histotype.

Some studies have shown that there is a link between VDR levels and recurrence of breast cancer, tumour size, and death from breast cancer as recently reported in melanoma [40]. This means that appropriate levels of VDR could exert positive effects on cancer outcome [41] and could have important implications for designing novel forms of therapy based on treatment with vitamin D or with vitamin D analogs [42].

However, the relationship between breast cancer and vitamin D is complex, not fully understood, and is still being studied [43–45]. These relationships could be also explained in terms of different *Cdx2* genotype status. For this reason, and driven by our results on the cell lines, we performed additional experiments among *Cdx2* polymorphism and estrogen receptor ER expression in different breast cancer subtypes. To our knowledge, our study is the first to explore the distribution of this polymorphism in BC cell lines and, in parallel, in BC tissues classified according to molecular subtypes. When we consider patients according to BC molecular subtypes, we did not observe any association between subtypes and VDR expression, but we found a significant association with *Cdx2* genotypes. In fact, our results indicated that BC in patients with variant homozygote AA presented bio-pathological characteristics more frequently associated with more aggressive tumours, such as HER2 subtype, and Triple negative. In contrast, variant GG seemed to be associated with BC bearing a luminal phenotype characterized by the expression of hormonal receptors and HER2 negativity.

Our findings seem to suggest that the *Cdx2* polymorphism could be involved in a biological pathway leading to a more aggressive phenotype. The use of polymorphism information may offer opportunities for identifying individuals, who will benefit of vitamin D treatment. These data are remarkable in that they might be of clinical relevance for the identification of risk groups, that could benefit chemoprevention protocols based on the anticancer genetic effects of vitamin D treatment.

In summary, our clinical findings revealed a close association between specific VDR *Cdx2* polymorphism and breast cancer bearing more aggressive phenotype. Moreover, *in vitro* observations established a different breast cancer cell sensitivity to vitamin D treatment on the base of their VDR *Cdx2* status. Together, these two new findings may provide an additional genetic marker that may be clinically useful in deciphering an individual’s response to vitamin D treatments.

Further studies are needed to clarify the functional aspects with respect to impact on VDR activity.
